# Thicker Shell Eggs with Enriched N-3 Polyunsaturated Fatty Acids and Lower Yolk Cholesterol Contents, as Affected by Dietary Nettle (*Urtica* *cannabina*) Supplementation in Laying Hens

**DOI:** 10.3390/ani10111994

**Published:** 2020-10-29

**Authors:** Jize Zhang, Ta Na, Yanmei Jin, Xiaoqing Zhang, Hui Qu, Qian Zhang

**Affiliations:** 1 Institute of Grassland Research, Chinese Academy of Agricultural Sciences, Hohhot 010010, China; zhangjize@caas.cn (J.Z.); tana_1980@163.com (T.N.); qhyulin@hotmail.com (H.Q.); zhangqian05@caas.cn (Q.Z.); 2 Marine College, Shandong University, Weihai 264209, China; jinym2001@sohu.com

**Keywords:** *Urtica cannabina*, n-3 polyunsaturated fatty acids, egg quality, cholesterol

## Abstract

**Simple Summary:**

The nettle *Urtica* *cannabina* (*U*. *cannabina*) belongs to the Urticaceae (nettle) family in the major group Angiosperms (flowering plants); it is distributed worldwide in temperate regions. While it was previously considered to be a weed, increasing evidence suggests that it is a valuable nutrient resource and has multiple biological functions when included in both human and animal diets. *U*. *cannabina* was given to laying hens in this experiment to study its effect on the hens’ performance, egg quality, yolk fatty acids composition, and serum biochemical parameters. The results revealed that dietary supplementation with *U*. *cannabina* has the potential to produce high-quality eggs. The data collected in this experiment can be used to inform further use of this plant to improve the lipid profile and fatty acid composition of eggs produced by hens fed on *U*. *cannabina*.

**Abstract:**

The nettle *Urtica* *cannabina* (*U.* *cannabina*) is not only a functional edible food for humans but also a potential alternative feed resource for poultry, providing protein, fatty acids, vitamins, and minerals. The present study was conducted to evaluate the effects of dietary *U.* *cannabina* on the production of high-quality eggs with enriched n-3 polyunsaturated fatty acids (PUFA) and lower cholesterol contents. One hundred and twenty laying hens were assigned to three groups [control, 15% alfalfa meal supplementation (TRTA), and 15% *U.* *cannabina* supplementation (TRTU)]. The results showed that the feed intake, yolk color, and shell thickness were increased (*p* < 0.05) in the *U.* *cannabina* group. Moreover, cholesterol contents of the yolk and serum were lower in the *U.* *cannabina* group (*p* < 0.05). The total n-3 PUFA concentration in the yolk was increased while the ratio of n-6/n-3 was reduced in the *U.* *cannabina* group (*p* < 0.01). In conclusion, the dietary inclusion of *U.* *cannabina* increased shell thickness, yolk n-3 PUFA levels, and yolk color, and reduced cholesterol contents of the yolk and serum without any negative impacts on health or laying performance.

## 1. Introduction

With the rapid development of the global population and living standards, there is an increasing demand for livestock and poultry production, which had led to increased requirements for livestock and poultry feedstuffs. Over the last decade, the shortage of traditional poultry feedstuffs and the increased price of these has provided significant challenges and has inspired researchers to seek out unconventional or alternative feedstuffs. Alternative feed ingredients have garnered increased attention intending to develop a more sustainable, environmentally friendly, and economically viable poultry feed to reduce the current dependency on imported feeds and to reduce the costs associated with the production of high-quality poultry products [1].

The nettle *Urtica cannabina* (*U*. *cannabina*) belongs to the Urticaceae (nettle) family in the major group Angiosperms (flowering plants); it is distributed worldwide in temperate regions [2]. Previously, nettle was considered a weed due to its soil coverage and rapid growth. However, the economic and ecological advantages of cultivating nettle are numerous. For example, nettle cultivation can improve soils over-fertilized with nitrogen and phosphate, reduce soil heavy metal content, and promote the biodiversity of local flora and fauna [3]. In addition to these benefits, nettle is a rich source of nutrients, such as proteins and fatty acids (especially α-linolenic, cis-9,12-linoleic, and palmitic acids), it is well balanced in amino acids, and is an abundant source of minerals and vitamins [4,5]. It also contains many biologically active compounds in its roots and leaves, such as lignanoids, triterpenoids, steroids, polysaccharides, and isolectins, which have been shown to possess anti-inflammation activities in vitro [6]. The young nettle leaf and stem are edible and thus, are commonly consumed as a food and are also used medicinally for various diseases [7]. Previous research has demonstrated that mice fed a diet supplemented with nettle plants exhibited significant reductions in lipid metabolism, with reductions in triglycerides and total cholesterol contents in blood plasma [8]. In monogastric animals, the composition of fatty acids stored in adipose tissue, muscle, or animal products such as eggs largely reflects the ingested lipids [9]. The growing market demand for designed eggs with lower cholesterol and higher n-3 polyunsaturated fatty acids is gaining increased attention and a number of relevant studies have been conducted. In these studies, unconventional feedstuffs such as rubber seeds and sugar beet pulp, etc., have been found to produce specialty eggs and reduced feed costs [10,11]. Furthermore, organic egg production requires that poultry have access to forage materials, for instance, alfalfa, red clover, or nettle plants [12,13].

To date, most studies of nettle as an additive in poultry feeds have examined the growth and slaughter performance of broilers and their antioxidation and immune function [14,15,16]. Limited data are available on the use of *U*. *cannabina* for laying hens. In particular, the studies have examined the effects of dietary nettle supplementation, as an alternative ingredient in poultry feeds, on vitamin and total cholesterol contents, and egg yolk fatty acid composition [13]. Therefore, the aim of this study was to determine the effects of dietary *U*. *cannabina* supplementation on laying performance, egg quality, fatty acid composition, and cholesterol contents of yolks, and to assess the value of *U*. *cannabina* as a dietary ingredient for the production of high-quality eggs.

## 2. Materials and Methods

The present experiment was conducted according to the Regulations for the Administration of Affairs Concerning Experimental Animals of the State Council of the People’s Republic of China. The research protocol was approved by the Committee on Experimental Animal Management of the Chinese Academy of Agricultural Sciences (Beijing) (approval no. 66/17.05.2019).

### 2.1. Forage Preparation

*U. cannabina* was harvested at the early flowering stage (approximately 10% flowering) in early July 2019 on grassland in Xilingol (Inner Mongolia, China), where the average temperature is between 15 to 20 °C from June to August. Mechanically harvested *U. cannabina* was dried naturally in the field. Alfalfa was purchased from Caodu Co., Ltd. (Xilingol, Inner Mongolia). It was cut and collected by a machine in mid-July and then dried naturally. All forage was grounded to powder mechanically for further diet preparation.

### 2.2. Experimental Design, Dietary Treatment, and Management

The experiment was performed at a private farm (Xilingol, Inner Mongolia). A total of 120 28-week-old Hy-line brown laying hens were randomly assigned to three treatments for a period of 8 weeks. Each treatment (40 hens) was further randomly divided into five replicates. The hens were kept in cages (each cage included two hens) equipped with a nipple drinker and trough feeders under 16 h of light per day until the end of the experiment. Four cages with the same diet trough were arranged sequentially as a replicate. The average temperature was 24 ± 2 °C in the laying hens’ house during the experimental period.

The diets were formulated to meet the recommendations of the National Research Council (NRC), 1994 for laying hens. The alfalfa meal and *U*. *cannabina* were supplemented in control diets (corn-soybean meal diets) as partial substitutions for yellow corns and soybeans. The experimental diets of the groups were as follows: Control, 15% alfalfa meal supplementation (TRTA), and 15% *U*. *cannabina* meal supplementation (TRTU) (Table 1).

### 2.3. Hen Performance and Egg Quality

All laying hens were separately weighed at the start and end of the study. Egg production and egg weights were measured each day. From 28 to 36 weeks of age, the feed intake was recorded weekly while the egg number and egg weights were recorded for each replicate each week. The feed conversion ratio was calculated as grams of feed intake/grams of egg weight, and egg mass was calculated as egg weight × percentage of egg production. Weights of the albumen, yolk, and shell (eight eggs per replicate) were recorded separately on the last 3 days of the 2nd, 4th, 6th, and 8th week during the experiment. Egg quality parameters were determined including shell thickness, egg yolk color, and Haugh units. Shell thickness was measured using a vernier caliper and was taken as the mean value at three points: The two narrow ends and in the middle of the egg. Yolk color and Haugh unit (as an interior egg quality indicator) were determined using an egg multi-tester (EMT-7300, Robotmation, Tokyo, Japan). The egg shape index was measured as previously described [11,17].

### 2.4. Egg Chemical Composition Analysis

During the 8th week of the study, 40 eggs per treatment (eight eggs/replicate) were collected to analyze the proximate chemical composition, total cholesterol content, and fatty acid composition of the yolk. Among those, 60 eggs (20 eggs/treatment) were chosen for chemical analysis, including moisture, crude protein (CP) (N × 6.25), ether extract (EE), and ash content, using methods developed by the AOAC (Association of Official Analytical Chemists) [18]. The remaining 60 eggs (20 eggs per treatment) were characterized for biochemical parameters. The egg yolk total cholesterol content (mg/g) was measured by high-performance liquid chromatography (HPLC) developed by Wen et al. [11]. The fatty acid composition of the yolk was analyzed using an Agilent 7890A gas chromatography (Agilent Technologies, Wilmington, USA) according to the Chinese National Standards of GB/T 22223-2008. For yolk vitamin analysis, vitamins A and B_2_ were determined using HPLC, as described in the previous research [19,20]. The concentrations of mineral elements in eggs, such as P, Ca, Fe, Zn, Mn, and Se, were analyzed by a Varian ICP-optical emission spectrometer (720-ES) (Varian Medical Systems, Palo Alto, USA) developed by Attia et al. [21].

### 2.5. Blood Biochemical Measurements

At the end of the experiment, blood samples were taken from hens via the wing vein of hens (two birds/replicate, 10/treatment). The serum was obtained by centrifugation twice at 2000 g at 4 °C for 30 min and then at 400 g at 4 °C for 10 min. The serum was then stored at −20 °C until analysis. For analysis of the biochemical parameters of the serum, the concentrations of total protein, albumin, globulin, total cholesterol, triglyceride, high-density lipoprotein-cholesterol (HDL-cholesterol), low-density lipoprotein-cholesterol (LDL-cholesterol), alanine aminotransferase (ALT), and aspartate aminotransferase (AST) were measured by an automatic biochemistry analyzer (Hitachi, Tokyo, Japan).

### 2.6. Statistical Analysis

Data on the performance and egg quality traits (except yolk color and yolk cholesterol content) of the laying hens in each treatment at the 2nd, 4th, 6th, and 8th week were submitted to the repeated measures analysis using the MIXED procedure of SAS. The following model was used:

*Y_jki_* = *μ* + *A_k_* + *B_i_* + *AB_ki_* + *e_jki_*, (1)

where *Y_jki_* is the target variable, *μ* is the overall mean, *A_k_* is the fixed effect of the treatment, *B_i_* is the fixed effect of the week, *AB_ki_* is the interaction effect of the treatment × week, and *e_jki_* is the residual error. Differences between the means of the treatments and weeks were compared by Duncan’s multiple range test. The yolk color and cholesterol content, egg chemical compositions, and blood biochemical parameters were analyzed by ANOVA as a completely randomized design, with a model that included treatment effects and experimental error. Individual animals were considered as experimental units. When the ANOVA was significant (*p* < 0.05), mean differences were analyzed by Duncan’s multiple range test.

## 3. Results

### 3.1. Laying Production Performance

The effect of dietary supplementation of *U. cannabina* on the performance of laying hens is presented in Table 2. The daily feed intake and egg production were increased in the *U. cannabina* group compared with the control group (daily feed intake, *p* < 0.001; egg production, *p* < 0.05); these two performance parameters were also significantly affected by the duration of supplementation (*p* < 0.001). The dietary *U. cannabina* supplementation and duration of supplementation had no effects on egg weight, egg mass, or the feed conversion ratio. There was a slightly higher average egg weight and egg mass in the *U. cannabina* group compared to the control and alfalfa groups across the whole experimental period (28–36 weeks of age), but these differences were not statistically significant.

### 3.2. Egg Quality Traits

**Figure 1:**
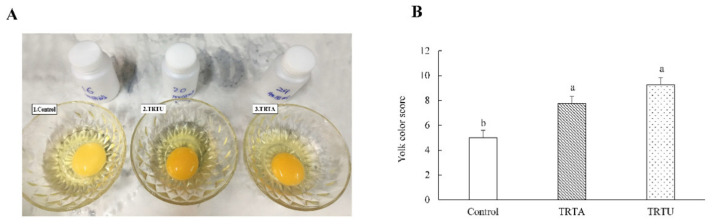
Egg yolk color (**A**) and statistical analysis of egg yolk color (**B**) of laying hens (36 weeks of age) fed diets containing alfalfa (TRTA) or *U. cannabina* (TRTU). Data points are group means ± SEM. a,b Denotes significant (*p* < 0.05) differences among the treatments.

As shown in Table 3, none of the egg quality traits (egg shape index, yolk percentage, albumen percentage, and Haugh unit), except for shell percentage and eggshell thickness, were affected by the dietary *U. cannabina* supplementation or duration of supplementation. The shell percentage and eggshell thickness of eggs from laying hens fed *U. cannabina* were higher than those from birds fed the control diet or alfalfa (shell percentage, *p* < 0.05; eggshell thickness, *p* < 0.001). In addition, the above two parameters increased with the duration of supplementation (shell percentage, *p* < 0.05; eggshell thickness, *p* < 0.001). The egg yolk color score was improved in the *U. cannabina* group compared to the control group (*p* = 0.001) (Figure 1). The score of the *U. cannabina* group was slightly higher than that of the alfalfa group, though this difference was not statistically significant.

### 3.3. Egg Chemical Composition

At the end of the experiment, there were no differences in CP% or EE% of eggs from the treatment groups. On the other hand, the vitamin contents (vitamin A and vitamin B2) and ash% of eggs laid by hens receiving the diet containing *U. cannabina* were greater than those of hens in the control and alfalfa groups (ash and vitamin B2, *p* < 0.01; vitamin A, *p* < 0.05) (Table 4). All mineral element contents (except Se), especially Ca and Mn contents, of eggs from hens fed the *U. cannabina* diet were significantly higher compared with both the control and alfalfa diet groups (Ca, *p* = 0.004; Mn, *p* = 0.007) (Table 4).

### 3.4. Egg Yolk Lipid Profile and Fatty Acids Composition

Eggs from laying hens fed the *U. cannabina* diet had lower yolk cholesterol contents compared to eggs from hens fed the control diet (Table 5). The fatty acids profile of the egg yolks is shown in Table 5. The dietary supplementation with *U. cannabina* did not affect the contents of individual or total saturated (SFA) or n-6 polyunsaturated fatty acids (n-6 PUFA). There was a significant reduction in total monounsaturated fatty acids (MUFA) in the group supplemented with *U. cannabina* (*p* = 0.007); specifically, the content of oleinic acid was decreased by 12.17% (*p* = 0.013) in the *U. cannabina* group compared to the control group. Further, *U. cannabina* had a significant effect on the contents of total n-3 PUFA (*p* = 0.009), α-linolenic acid (ALA) (*p* = 0.029), eicosapentaenoic acid (EPA) (*p* = 0.018), and docosahexaenoic acid (DHA) (*p* = 0.018). Due to the higher content of total n-3 PUFA in the *U. cannabina* group, the ratio of n-6/n-3 PUFA was more favorable in the *U. cannabina* group (*p* = 0.001). The smallest and most favorable ratio of 4.92 was found in the *U. cannabina* group, followed by 6.25 in the alfalfa group, and 12.67 in the control group.

### 3.5. Blood Biochemical Parameters

Table 6 shows the effects of dietary *U. cannabina* supplementation on blood biochemical parameters in 36-week-old laying hens. The serum levels of total protein (*p* = 0.015), globulin (*p* = 0.005), and HDL-cholesterol were higher in the *U. cannabina* group than the control group. The serum levels of albumin/globulin (*p* = 0.004), total cholesterol (*p* = 0.034), and triglycerides (*p* = 0.010) were significantly reduced in the *U. cannabina* group compared to the control group. There were no differences in serum levels of albumin, AST, ALT, and LDL-cholesterol among the three treatments.

## 4. Discussion

In general, nettle plants are considered to be highly nutritional foods with multiple functional values [22], including the potential to lower serum cholesterol in animals [8]. However, there is only limited research on the effects of dietary *U. cannabina* supplementation of laying hens. Thus, the purpose of the current research was to investigate whether the performance, egg quality traits, egg yolk lipid profile, and blood biochemical parameters of laying hens were affected by the dietary supplementation of *U. cannabina*. The obtained results indicate that the addition of this unconventional feedstuff to the diets of laying hens can contribute to the production of high-quality eggs.

*U. cannabina* had no adverse effect on the overall production performance of hens in the present study. To date, the effect of nettle plants in poultry diets on the performance of laying hens has been a subject of debate. For instance, previous studies have reported that feed intake remained unchanged [23,24] or decreased upon dietary *Urtica dioica* (*U. dioica*) supplementation [25]. Further, the feed conversion ratio has been reported to remain the same [23] or even increased in response to the *U. dioica* supplementation [26,27]. The primary terpenoids in *U. dioica* (especially carvacrol and carvone) possess multiple biological functions, including growth promotion and antioxidant functions [28]. It has been demonstrated that differential geographical distribution and environmental conditions influence these compounds and their derivatives, causing differences in their content in plants [29]. This may be one reason for the discrepancies in the literature. Other factors such as the strain of birds studied (broiler, laying hen, or quail) and their physiological status may contribute to the varied findings.

In the current study, the inclusion of *U. cannabina* in the diet of laying hens was associated with increased shell thickness and shell percentage. On the contrary, previous research reported that the inclusion of *U. dioica* in the diets of 70-week-old Brown Nick laying hens and Japanese quails did not influence egg quality characteristics such as shell thickness, Haugh unit, and egg composition (albumen, yolk, and shell) [23,30]. Kregiel et al. [3] reported that in addition to the large amounts of calcium in nettle leaves and stem, nettle leaves also have an abundance of zinc, magnesium, and iron. Enrichment of egg mineral elements was observed in the *U. cannabina* group in the present study, which is consistent with the above results.

Moreover, previous research reported that the systemic administration of *U. dioica* increased serum calcium concentration and bone volume and accelerated new bone formation due to its anti-inflammatory effect in rats [31,32]. The anti-inflammatory effect of *U. dioica* was mainly attributed to its antioxidant constituents, especially the phenolic compounds, which inhibit nuclear factor (NF)-κB activation, thereby reducing pro-inflammatory gene products [32]. Furthermore, as an important eggshell formation factor in the avian uterus, calbindin plays a primary role in Ca^2+^ transportation [33]. The expression of calbindin mRNA was found to be disturbed in the uteri of birds infected with the avian influenza virus via the influences of substances from cytotoxic cells and proinflammatory cytokines; this was found to produce a deterioration of eggshell quality [34,35]. Therefore, based on the presence of abundant calcium in the eggs from the *U. cannabina* group in this study, it can be assumed that supplementation with *U. cannabina* may increase the calcium transport by calbindin due to its anti-inflammatory effect, thus increasing shell calcification which consequently leads to improvements in shell quality features. However, the related gene expressions and concentrations of cytokines still require further research.

The yolk color also represents a vital indicator of egg quality; this quality is particularly important to consumers. In our study, the enhanced yolk color was found in the *U. cannabina* group. A similar phenomenon was also observed in another nettle study. Loetscher et al. [30] studied the effect of *U. dioica* on egg quality traits and found that yolk yellowness (b*) was increased in the *U. dioica* group, depending on the dosage. Further, *U. dioica* was found to be equally as effective as synthetic pigmentation. Nettle is rich in yellow-colored xanthophylls, with lutein being the predominant compound, followed by β-carotene and zeaxanthin [3]. It has been shown that the inclusion of *U. dioica* in food increases the enrichment and bioaccessibility of lutein and *β*-carotene (provitamin A carotenoids) at the duodenum digestion stage [22]. Furthermore, xanthophylls were found to be absorbed in the digestive tract and deposited in subcutaneous fat and yolk leading to higher yolk color scores [11]. Therefore, the higher yolk color observed in the *U. cannabina* group in this study might be due to its high levels of effective polar xanthophylls, such as lutein and zeaxanthin, which could be deposited in egg yolks [36]. In addition, β-carotene is almost completely converted into vitamin A in poultry [36]. This is consistent with the observation of increased vitamin A content in the *U. cannabina* supplementation group in the present study.

Recently, designed eggs with improved lipid profiles, such as lower cholesterol and higher PUFA, have gained popularity among consumers. In the present study, the concentration of egg yolk cholesterol was decreased in the *U. cannabina* supplementation group compared to the control group. These findings are consistent with the findings of Moula et al. [23] in which dietary supplementation with the *U. dioica* powder reduced the total cholesterol in the yolks of eggs from Japanese quails. Nettle plants have been shown to be rich in bioactive compounds such as phytosterol, pentacyclic triterpenoids, coumarins, and ceramides [3]. The lower egg yolk cholesterol concentration in the *U. cannabina* group can be attributed to its phytosterol components (such as astigmasterol and campesterol compounds) which can decrease the cholesterol concentration in micelles to reduce the absorption of cholesterol in the gut, thereby lowering cholesterol levels in the blood [8], and subsequently, in animal products.

Nettle leaves are rich sources of essential fatty acids. ALA is the pre-dominant fatty acid, accounting for 40.7% of the fatty acids in the mature leaves [5]. The composition of fatty acids stored in monogastric animals largely indicates that the lipid and fatty acid composition of poultry eggs can be altered by diet [37]. Hence, eggs can be an important source of n-3 PUFA when laying hens are fed a diet containing a high level of n-3 PUFA [11,38]. Numerous fatty acid desaturases play key roles in synthesizing PUFA. Several desaturases are absent in animals and humans, such as delta-12 and delta-15 desaturases [39]. Thus, ALA must be obtained from the diet; it can be converted into EPA and DHA by delta-6 desaturase catalyzed dehydrogenation and the addition of two carbons by an elongase [40]. An n-3 PUFA-enriched diet was found to increase the expression level of delta-6 desaturase mRNA [11]. In the present study, elevation of DHA and EPA contents was also considered to reflect primary dietary regulation of delta-6 desaturase gene. In addition, the *U. cannabina* supplementation in the current study was associated with a reduction in the MUFA (predominantly C18:1n9c) proportion and a significant elevation in the ALA proportion compared to the control diet. This phenomenon is consistent with a previous study where hens fed with rubber seeds oil (an n-3 PUFA enriched ingredient) were found to lay eggs with a decreased content of C18:1n9 along with an increased ALA content; these findings were thought to be related to the conversion of C18:1n9 into ALA through desaturation [11]. In our study, the content of n-3 PUFA was significantly higher in the *U. cannabina* group than in the control group (*p* = 0.009), which caused a great improvement in the ratio of n-6/n-3 in the *U. cannabina* group (4.95 vs. 12.67), exceeding the recommended intake ratio of 4:1 (n-6 PUFA 10 g/d, n-3 PUFA 2.5 g/d) by the European Food Safety Authority (EFSA) [41]. Stojčić et al. [14] similarly reported that dietary supplementation with fresh nettle reduced the ratio of n-6/n-3 and increased the proportion of n-3 PUFA in the breast meat of broiler. It is important to maintain a low ratio of n-6/n-3 in the diet due to its role in lowering the risk of cardiovascular diseases [42,43]. A higher total n-3 PUFA content, especially DHA, in the group supplemented with *U. cannabina* could be explained by the protective function of antioxidative compounds such as lutein, provitamin A, tocopherol, and phenolic compounds. Stinging nettle is an abundant source of lutein, which has been considered to be an important natural pigment for improving egg yolk and broiler skin color [15,30]. Lutein and phenolic compounds (particularly flavonoids) are potent antioxidants due to their free radical quenching activities [32,44]. Grčević et al. [38] reported that the addition of marigold powder (rich in lutein) to the laying hens’ feed significantly increased egg lutein content and preserved the high content of DHA in the yolks. Loetscher et al. [15] provided broilers with a diet supplemented with 2.5% *U. dioica* and found that the tocopherols content of the breast meat was significantly increased. Based on these results, we assume that the richness of antioxidants in *U. cannabina* had an impact on the conservation of DHA in the lipids of the egg yolks by protecting DHA from oxidation and damage, thereby maintaining a higher level of DHA in the *U. cannabina* group. Further studies should determine the level of these antioxidants in both *U. cannabina* and the eggs produced by hens fed the *U. cannabina* supplemented diet. Nonetheless, these findings indicate that, as an important n-3 PUFA source, *U. cannabina* contributed to the production of n-3 PUFA-enriched and stable eggs.

In the current study, the laying hens fed with *U. cannabina* exhibited no health issues and normal serum biochemical parameters were observed. Hepatic serum levels of ALT and AST are frequently used to evaluate liver injuries. In this study, there were no significant changes in serum ALT and AST levels in the *U. cannabina* group compared with the control group, which confirms that there was no diet-induced liver injury [37]. A decrease in total cholesterol and triglycerides and an increase in HDL-cholesterol was observed in the serum of hens fed with *U. cannabina* due to the rich sterols content of *U. cannabina*. On the other hand, a higher HDL-cholesterol content in the *U. cannabina* group facilitated the translocation of cholesterol from the peripheral tissues to the liver for catabolism [8], which might be another reason for the reduced serum level of cholesterol.

## 5. Conclusions

Overall, the results of this study offer important information on the egg production and physiological responses of laying hens fed with *U. cannabina*. These findings support the potential application of *U. cannabina* as a dietary supplementation for laying hens. Moreover, the dietary *U. cannabina* supplementation had beneficial effects on blood biochemical parameters and egg chemical composition, especially less total cholesterol, enriched n-3 PUFA, and vitamin A, leading to healthy eggs that will be desirable to consumers.

## Figures and Tables

**Table 1:** Nutrient composition of alfalfa and *U. cannabina*, and the composition and nutrient levels of experimental diets for laying hens (based on dry matter).

Items	Control	TRTA	TRTU	Alfalfa	*U. cannabina*
Ingredients (%)					
Yellow corn	61.0	58.6	60.0	-	-
Soybean meal	19.7	16.3	13.8	-	-
Canola oil	1.40	1.40	1.40	-	-
Alfalfa meal	0	15.0	0	-	-
*U. cannabina* meal	0	0	15.0	-	-
Corn gluten	7.90	-	2.50	-	-
Limestone	8.00	6.90	5.70	-	-
Dicalcium phosphate	1.20	1.00	0.800	-	-
Premix ^1^	0.600	0.600	0.600	-	-
Lysine	0.100	0.100	0.100	-	-
DL-Methionine	0.100	0.100	0.100	-	-
Total	100	100	100	-	-
Calculated values					
ME (MJ/kg)	11.5	11.3	11.5	6.55	6.82
Crude protein (%)	16.0	16.0	16.0	20.5	25.6
Calcium (%)	3.54	3.41	3.42	2.56	6.04
Total phosphorus (%)	0.620	0.530	0.550	0.210	0.670
Methionine (%)	0.410	0.400	0.400	0.200	0.240
Lysine (%)	0.890	0.860	0.900	0.780	1.40
NDF (%)	11.3	11.8	11.8	33.1	25.0

TRTA: Alfalfa; TRTU: *U. cannabina*; ME: Metabolizable energy; NDF: Neutral detergent fiber. ^1^ Supplied per kilogram of diet as detailed in Wen et al. [11] with the following slight modification: Vitamin B_1_, 2 mg; vitamin B_2_, 6 mg; vitamin B_6_, 5 mg; vitamin D_3_, 3000 IU vitamin K_3_, 3 mg.

**Table 2:** Effects of dietary *U. cannabina* supplementation on laying hen production performance.

Items	Treatments			Time on Feed		SEM	*p*-Value		
	Control	TRTA	TRTU	32 Weeks	36 Weeks		TRT	T	TRT × T
Daily feed intake (g)	109 ^b^	114 ^a^	115 ^a^	111 ^b^	115 ^a^	0.345	<0.001	<0.001	0.943
Egg production (%)	93.4 ^b^	95.5 ^ab^	95.6 ^a^	95.8 ^a^	90.9 ^b^	0.598	0.049	<0.001	0.058
Egg weight (g)	57.4	57.6	58.2	57.7	58.1	0.143	0.060	0.362	0.717
Egg mass per day (g)	53.6	55.0	55.7	54.3	55.4	0.409	0.058	0.204	0.924
Feed conversion ratio (g feed/g egg)	2.03	2.08	2.06	2.06	2.09	0.017	0.226	0.413	0.906

^a, b^ Means in the same row with different superscript letters indicate difference (*p* < 0.05); SEM: Standard error of the mean; TRTA: Alfalfa; TRTU: *U. cannabina*; TRT: Treatments; T: Time on feed (28 to 36 weeks of age).

**Table 3:** Effects of dietary *U. cannabina* supplementation on the egg quality of laying hens.

Items	Treatments			Time on Feed		SEM	*p*-Value		
	Control	TRTA	TRTU	32 Weeks	36 Weeks		TRT	T	TRT × T
Egg shape index	1.30	1.29	1.27	1.29	1.29	0.060	0.276	0.897	0.764
Yolk (%)	26.8	25.6	26.3	25.8	26.6	0.003	0.152	0.126	0.622
Shell (%)	14.1 ^c^	14.6 ^b^	15.4 ^a^	14.2 ^b^	15.1 ^a^	0.095	0.023	0.016	0.035
Albumen (%)	59.0	59.8	58.3	59.1	59.2	0.569	0.262	0.379	0.661
Eggshell thickness (mm)	0.340 ^c^	0.361 ^b^	0.381 ^a^	0.347 ^b^	0.374 ^a^	0.005	0.001	0.001	0.018
Haugh unit	80.1	80.5	81.1	80.8	80.6	1.52	0.135	0.438	0.927

^a–c^ Means in the same row with different superscript letters indicate differences (*p* < 0.05); SEM: Standard error of the mean; TRTA: Alfalfa; TRTU: *U. cannabina*; TRT: Treatments; T: Time on feed.

**Table 4:** Effects of dietary *U. cannabina* supplementation on the chemical composition of eggs from laying hens.

Item	Treatment			SEM	*p*-Value
	Control	TRTA	TRTU		
CP (%)	11.7	12.4	13.8	0.409	0.084
EE (%)	8.10	8.41	6.59	0.500	0.321
Ash (%)	0.726 ^b^	0.856 ^b^	1.44 ^a^	0.117	0.002
Vit A (µg/100 g)	190 ^b^	192 ^b^	223 ^a^	5.55	0.012
Vit B2 (mg/100 g)	0.322 ^b^	0.353 ^b^	0.484 ^a^	0.020	0.003
P (mg/kg)	1541 ^b^	2207 ^a^	2445 ^a^	163	0.029
Ca (mg/kg)	321 ^c^	542 ^b^	651 ^a^	50.3	0.004
Fe (mg/kg)	20.4 ^b^	25.9 ^b^	34.9 ^a^	2.44	0.015
Zn (mg/kg)	14.1 ^b^	14.3 ^b^	18.8 ^a^	0.929	0.036
Mn (mg/kg)	1.76 ^b^	1.89 ^b^	2.39 ^a^	0.106	0.007
Se (µg/100 g)	2.83	2.92	4.83	0.451	0.108

^a–c^ Means in the same row with different superscript letters indicate differences (*p* < 0.05); SEM: Standard error of the mean; TRTA: Alfalfa; TRTU: *U. cannabina*; CP: Crude protein; EE: Ether extract; Vit: Vitamin.

**Table 5:** Effects of dietary *U. cannabina* supplementation on the total cholesterol content and fatty acid composition (g/100 g FA) in eggs from laying hens.

Item	Treatment			SEM	*p*-Value
	Control	TRTA	TRTU		
Total cholesterol (mg/100 g) (egg yolk)	15.4 ^a^	15.2 ^ab^	13.5 ^b^	0.370	0.013
C14:0	0.503	0.662	0.671	0.043	0.204
C16:0	26.7	27.5	26.8	0.611	0.874
C17:0	0.133	0.145	0.136	0.012	0.935
C18:0	8.45	8.40	7.44	0.367	0.514
SFA	35.8	36.7	35.0	0.541	0.502
C14:1n9c	0.092	0.109	0.118	0.011	0.637
C16:1n9c	4.60	4.41	4.13	0.300	0.851
C18:1n9t	2.57	2.48	2.35	0.253	0.955
C18:1n9c (oleinic acid)	37.7 ^a^	35.7 ^ab^	33.1 ^b^	0.760	0.013
C20:1	0.240	0.239	0.267	0.019	0.798
MUFA	45.2 ^a^	42.9 ^ab^	40.0 ^b^	0.840	0.007
C18:2 (*trans*-9, *trans*-12)	2.38	2.31	2.77	0.407	0.910
C18:2n-6 (LA)	11.1	10.9	12.7	0.412	0.122
C20:2n-6	0.097	0.116	0.120	0.014	0.828
C20:4n-6 (AA)	2.51	2.34	2.89	0.213	0.624
n-6 PUFA	16.1	15.6	18.5	0.679	0.181
C18:3n-3 (ALA)	0.522 ^b^	0.675 ^a^	0.746 ^a^	0.040	0.029
C20:5 n-3 (EPA)	0.265 ^b^	0.354 ^b^	0.622 ^a^	0.062	0.018
C22:6n-3 (DHA)	0.502 ^b^	1.60 ^ab^	2.43 ^a^	0.324	0.018
n-3 PUFA	1.29 ^b^	2.63 ^a^	3.80 ^a^	0.407	0.009
n-6/n-3 PUFA	12.7 ^a^	6.25 ^b^	4.92 ^b^	1.26	0.001

TRTA: Alfalfa; TRTU: *U. cannabina*; SFA: Saturated fatty acid; MUFA: Monounsaturated fatty acid; LA: Linoleic acid; AA: Arachidonic acid; PUFA: Polyunsaturated fatty acid; ALA: α-Linolenic acid; EPA: Eicosapentaenoic acid; DHA: Docosahexaenoic acid. ^a, b^ Means in the same row with different superscript letters indicate differences (*p* < 0.05); SEM: Standard error of the mean.

**Table 6:** Effects of dietary *U. cannabina* supplementation on blood biochemical parameters of laying hens.

Items	Treatment			SEM	*p*-Value
	Control	TRTA	TRTU		
Total protein (g/dL)	4.23 ^b^	4.96 ^ab^	5.55 ^a^	0.163	0.015
Albumin (g/dL)	1.90	1.80	2.05	0.056	0.409
Globulin (g/dL)	2.32 ^b^	3.16 ^a^	3.50 ^a^	0.151	0.005
A/G	0.846 ^a^	0.586 ^b^	0.592 ^b^	0.042	0.004
ALT (U/L)	14.9	10.5	13.5	1.04	0.504
AST (U/L)	164	129	157	10.2	0.644
Total cholesterol (mmol/L)	4.02 ^a^	2.52 ^b^	2.79 ^b^	0.170	0.034
Triglycerides (mmol/L)	11.6 ^a^	9.84 ^ab^	8.50 ^b^	0.355	0.010
HDL-cholesterol (mmol/L)	0.617 ^b^	1.47 ^a^	1.26 ^a^	0.104	0.001
LDL-cholesterol (mmol/L)	1.16	1.35	1.17	0.095	0.860

TRTA: Alfalfa; TRTU: *U. cannabina*; ALT: Alanine aminotransferase; AST: Aspartate aminotransferase; A/G: Albumin/globulin; HDL: High-density lipoprotein; LDL: Low-density lipoprotein. ^a, b^ Means in the same row with different superscript letters indicate differences (*p* < 0.05); SEM: Standard error of the mean.
